# Physicians’ Perspectives on Collaboration with Community Pharmacists: Implications for Collaborative Care in Jordan

**DOI:** 10.3390/healthcare14152335

**Published:** 2026-08-01

**Authors:** Ameerah Hasan Ibrahim, May Almajawleh, Haneen Abuzaid, Bara’a Shawaqfeh, Razan Abujaber, Ruaa Hussien

**Affiliations:** Department of Pharmacy, Faculty of Pharmacy, Al-Zaytoonah University of Jordan, Amman 11733, Jordan; may.almajawleh@zuj.edu.jo (M.A.); haneen.abuzaid@zuj.edu.jo (H.A.); b.shawaqfeh@zuj.edu.jo (B.S.); r.abujaber@zuj.edu.jo (R.A.); raa9250076@ju.edu.jo (R.H.)

**Keywords:** community pharmacists, interprofessional collaboration, Jordan, patient care, physician attitudes, physician–pharmacist collaboration

## Abstract

**Background/Objectives**: Collaboration between physicians and community pharmacists (CPs) is important for ensuring high-quality healthcare delivery. However, evidence on physician–CP collaboration in Jordan remains limited, and no studies have specifically examined physicians’ perspectives on collaboration with CPs. This study aimed to explore physicians’ experiences and attitudes towards collaboration with CPs, their views on the impact of such collaboration on patient care, and their perceptions of CPs’ professional roles. **Methods**: Between January and March 2025, a cross-sectional survey was conducted among 380 physicians in Amman, Jordan, using convenience sampling. A five-section self-administered questionnaire was used to collect the data. Data were analysed using descriptive and inferential methods in SPSS version 28. **Results**: Of the 380 physicians directly invited to participate, 209 completed the questionnaire. All participants reported previous collaboration with CPs, although the frequency varied. Nearly half of the participants reported that collaboration occurred sometimes (47.4%, *n* = 99). Physicians perceived that CPs only sometimes had adequate clinical skills (59.3%, *n* = 124), confidence (61.2%, *n* = 128), or knowledge (65.6%, *n* = 137) to deliver effective patient care. Most physicians (>74%) demonstrated positive attitudes towards collaboration with CPs, particularly regarding communication, mutual respect, and shared goals in patient care. Nevertheless, neutral attitudes were reported towards trust in CPs’ professional decisions (42.1%, *n* = 88), meeting professional expectations (33.0%, *n* = 69), and therapeutic expertise (46.9%, *n* = 98). Almost all physicians agreed that collaboration with CPs could improve patient outcomes (98.1%, *n* = 205) and continuity of care (89.0%, *n* = 186). Physicians also expressed positive perceptions of CPs’ professional roles, mainly in improving medication adherence (91.9%, *n* = 192). Significant overall differences in attitude (*p* = 0.035) and perception (*p* = 0.025) scores were observed according to physicians’ job description; however, post hoc analysis identified a significant pairwise difference only for perception scores between general practitioners and specialists. **Conclusions**: This study found that the majority of physicians reported previous collaboration with CPs and generally held positive views and attitudes towards collaborative practice. However, uncertainty regarding CPs’ expertise and decision-making abilities suggests the necessity of professional role clarification and trust-building. These findings should be interpreted in the context of a convenience sample of physicians practising in Amman, predominantly in the private sector, and should not be generalised to all physicians in Jordan. Future research exploring CPs’ perspectives on collaboration with physicians is needed to support the findings of this study.

## 1. Introduction

Healthcare systems are becoming more complex and continue to face growing resource limitations. As a result, several healthcare objectives can no longer be effectively achieved by a single healthcare professional (HCP) or organisation working independently [1]. Therefore, many healthcare organisations, including healthcare clinics and community pharmacies, are increasingly being encouraged to move away from isolated practice towards more collaborative and integrated models of care. Combining the knowledge, skills, and expertise of different HCPs, alongside the active involvement of patients and service users, is vital for delivering effective patient-centred care [1,2,3,4].

Patient safety is an essential component of healthcare [5]. Several measures are implemented to enhance and maintain patient safety, including the training of healthcare staff, standardised clinical procedures, incident reporting systems, the monitoring and management of medical errors, adherence to infection-control practices, and ensuring access to appropriate medications and medical equipment [6,7,8,9]. Among these measures, physician–pharmacist collaboration has emerged as an important strategy for improving the safety, quality, and accessibility of healthcare services [2,10,11,12]. Moreover, evidence has shown that such collaboration can improve patient health outcomes, increase job satisfaction among HCPs, enhance continuity of care, and support the better delivery of patient care [4,13,14].

Despite growing evidence supporting the benefits of physician–pharmacist collaboration, implementing this type of collaborative practice remains challenging. Several barriers have been identified, such as negative physician attitudes, uncertainty about pharmacists’ clinical knowledge and decision-making abilities, limited communication, negative patient perceptions, and logistical and financial constraints [2,15]. To support successful collaboration, several facilitating factors have been suggested, including increasing awareness and trust between HCPs, improving pharmacists’ clinical training, clearly defining professional roles and practice guidelines, enhancing public perceptions of pharmacists, improving access to patient records, and strengthening communication between HCPs [2,4,16,17,18,19]. Understanding these factors is essential for strengthening collaboration between physicians and community pharmacists (CPs) and may contribute to improved healthcare delivery and patient outcomes.

In Jordan, community pharmacy services are highly accessible to the public, with community pharmacies widely distributed across the country and many patients seeking healthcare advice from pharmacists before consulting a physician [20,21]. Within the Jordanian healthcare system, where healthcare services are delivered through the public and private sectors, effective communication and collaboration between physicians and CPs are important to support continuity of care and optimise medication management [21,22].

HCPs’ experiences, preferences, and perceived barriers to collaborative practice play an important role in shaping effective working relationships [2]. Several quantitative and qualitative studies have been conducted internationally to explore HCPs’ perspectives on physician–pharmacist collaboration [2,15,23,24]. However, evidence from the Middle East remains limited, with only a small number of studies examining collaborative practice between physicians and pharmacists in countries such as Iraq [25], Kuwait [2], Qatar [26], and the United Arab Emirates [15]. To date, no published studies in Jordan have explored physician–CP collaboration or physicians’ perspectives on collaboration with CPs. Understanding local perspectives on physician–CP collaboration is vital, as healthcare systems, professional roles, and practice environments may differ from those reported in other countries. Additionally, such evidence may support the development of effective collaborative practice models that can enhance patient-centred care. Therefore, this study aimed to explore physicians’ experiences and attitudes towards collaboration with CPs, their views on the impact of such collaboration on patient care, and their perceptions of CPs’ professional roles.

## 2. Materials and Methods

### 2.1. Study Design and Setting

Between January and March 2025, a cross-sectional survey was conducted among physicians practising in Amman, Jordan. Amman has a high density of healthcare facilities and community pharmacies, facilitating the inclusion of physicians from diverse practice settings. This study was reported according to the Strengthening the Reporting of Observational Studies in Epidemiology (STROBE) guidelines [27].

### 2.2. Participants and Sampling

Physicians who were registered with the Jordan Medical Association and practising in public or private healthcare facilities were eligible for inclusion in the study. A convenience sampling method was employed due to feasibility considerations. At the time of the study, the Jordan Medical Association had approximately 44,000 registered physicians. The required sample size was estimated using the Raosoft^®^ (Seattle, WA, USA) sample size calculator. A target sample size of 380 physicians was determined based on a 95% confidence level, a 5% margin of error, and an assumed response distribution of 50%. This calculation was used to guide participant recruitment and should not be interpreted as indicating population representativeness or a guaranteed margin of error, as convenience sampling was employed.

### 2.3. Data Collection

Following an extensive literature review on interprofessional collaboration and physician–pharmacist relationships [2,4,15,16,17,18,19,25,26,28,29], the research team developed a self-administered questionnaire (Supplementary File S1). The questionnaire consisted of five sections (Supplementary Sections A–E; Table 1). Supplementary Section A collected demographic information about the participating physicians. Supplementary Section B consisted of descriptive questions exploring physicians’ experiences of collaboration with CPs. The frequency of collaboration between physicians and CPs was assessed using a single descriptive ordinal question with five response categories (never, rarely, sometimes, frequently, and always). The frequency of communication was assessed separately using specific time-based response categories (never, daily, 2–3 times per week, once per week, once every two weeks, once per month, and other), together with questions regarding the methods and reasons for communication. Supplementary Section C comprised the validated “Attitudes Towards Collaboration Instrument for GPs” (ATCI-GP), originally developed by Van et al. [30], which was used without modification to assess physicians’ attitudes towards collaboration with CPs. Although originally developed for general practitioners (GPs), the ATCI-GP measures general attitudes towards physician–pharmacist collaboration, including communication, trust, mutual respect, shared goals, professional role clarity, and perceptions of pharmacists’ contributions to medication management, rather than profession-specific clinical activities. Therefore, it was considered appropriate for use across the different physician groups included in the present study. Supplementary Sections D and E were developed by the research team following the literature review to explore physicians’ views on collaboration with CPs and their perceptions of the professional roles of CPs. These sections were intended as exploratory item sets rather than validated psychometric scales. Therefore, the results from Supplementary Sections D and E should be interpreted as exploratory findings. To ensure a common understanding of the concept among participants, a definition of collaboration in patient care (“cooperatively working together, sharing responsibilities for solving problems and making decisions to formulate and carry out plans for patient care”) was provided at the beginning of the questionnaire (Supplementary File S1). For the purposes of this study, the term “CP” referred to an individual pharmacist practising in a community pharmacy rather than the community pharmacy as an organisational setting. Responses in Supplementary Sections C–E were measured using a five-point Likert scale ranging from 1 (strongly disagree) to 5 (strongly agree). The questionnaire was pilot tested among 16 physicians to assess face validity, clarity, and comprehensibility prior to data collection. Based on the feedback received during pilot testing, minor wording modifications were made to improve the clarity and comprehensibility of some questions.

Following informed consent, participants completed the questionnaire in English, either electronically or in paper-based format. As English is the language of medical education and professional practice in Jordan, no linguistic adaptation of the questionnaire was necessary. The majority of physicians were directly approached by the researcher (R.H.) and completed the paper-based questionnaire during visits to healthcare facilities. A smaller proportion of physicians completed the electronic questionnaire using Google Forms after receiving individual invitations via WhatsApp or Facebook Messenger. The electronic survey link was not publicly posted on social media platforms or groups. To minimise duplicate participation, physicians completed the questionnaire in either paper-based or electronic format, and electronic invitations were sent only to physicians who had not completed the paper-based questionnaire. Participation was voluntary and anonymous. Written informed consent was obtained from participants completing the paper-based questionnaire. For the online questionnaire, an electronic consent form was presented on the first page, and participants were required to provide electronic informed consent before proceeding to the questionnaire. No personally identifiable information was collected. Responses submitted through Google Forms were stored in a password-protected account and were accessible only to the research team.

### 2.4. Data Analysis

Statistical analyses were conducted using IBM SPSS software version 28 (IBM Corp., Armonk, NY, USA). Descriptive statistics were used to present the data as frequencies and percentages. Responses in Supplementary Sections C–E were scored using a five-point Likert scale (1 = strongly disagree to 5 = strongly agree). For each participant, a mean item score was calculated separately for the attitude (Supplementary Section C; 13 items), views (Supplementary Section D; 5 items), and perception (Supplementary Section E; 10 items) sections by averaging the responses across all items within each section. The median (IQR) of these participant-level mean scores was then reported and used in subsequent analyses. The theoretical score range for each section was 1 to 5, with higher scores indicating more positive attitudes, views, or perceptions. No item-level missing data were observed; therefore, all analyses were based on the full study sample (*N* = 209). No negatively worded items were included in the questionnaire; therefore, reverse coding was not required. Data normality was assessed for the participant-level mean scores of the attitude, views, and perception sections using the Kolmogorov–Smirnov and Shapiro–Wilk tests, together with visual inspection of histograms and normal Q–Q plots. As the participant-level mean scores were not normally distributed, continuous variables are reported as medians and interquartile ranges (IQRs). Comparisons between demographic variables and continuous outcomes were performed using the Mann–Whitney U test or Kruskal–Wallis test, as appropriate. For statistically significant Kruskal–Wallis tests, post hoc pairwise comparisons with adjustment for multiple comparisons were performed, and epsilon squared (ε^2^) was used to estimate the effect size. Effect sizes were interpreted as small (ε^2^ ≥ 0.01), medium (ε^2^ ≥ 0.06), and large (ε^2^ ≥ 0.14). Internal consistency of the attitude (Supplementary Section C), views (Supplementary Section D), and perception (Supplementary Section E) items was assessed using Cronbach’s alpha. A *p*-value of <0.05 was considered statistically significant.

A descriptive approach was taken to analyse the responses to the open-ended question [31]. All free-text responses were included in the analysis. The responses were reviewed several times by two members of the research team (A.H.I. and R.H.) to achieve a general understanding, and similar responses were grouped into broad categories to summarise the key findings. Any differences in categorisation were resolved through discussion and consensus.

## 3. Results

### 3.1. Participant Characteristics

**Figure 1:**
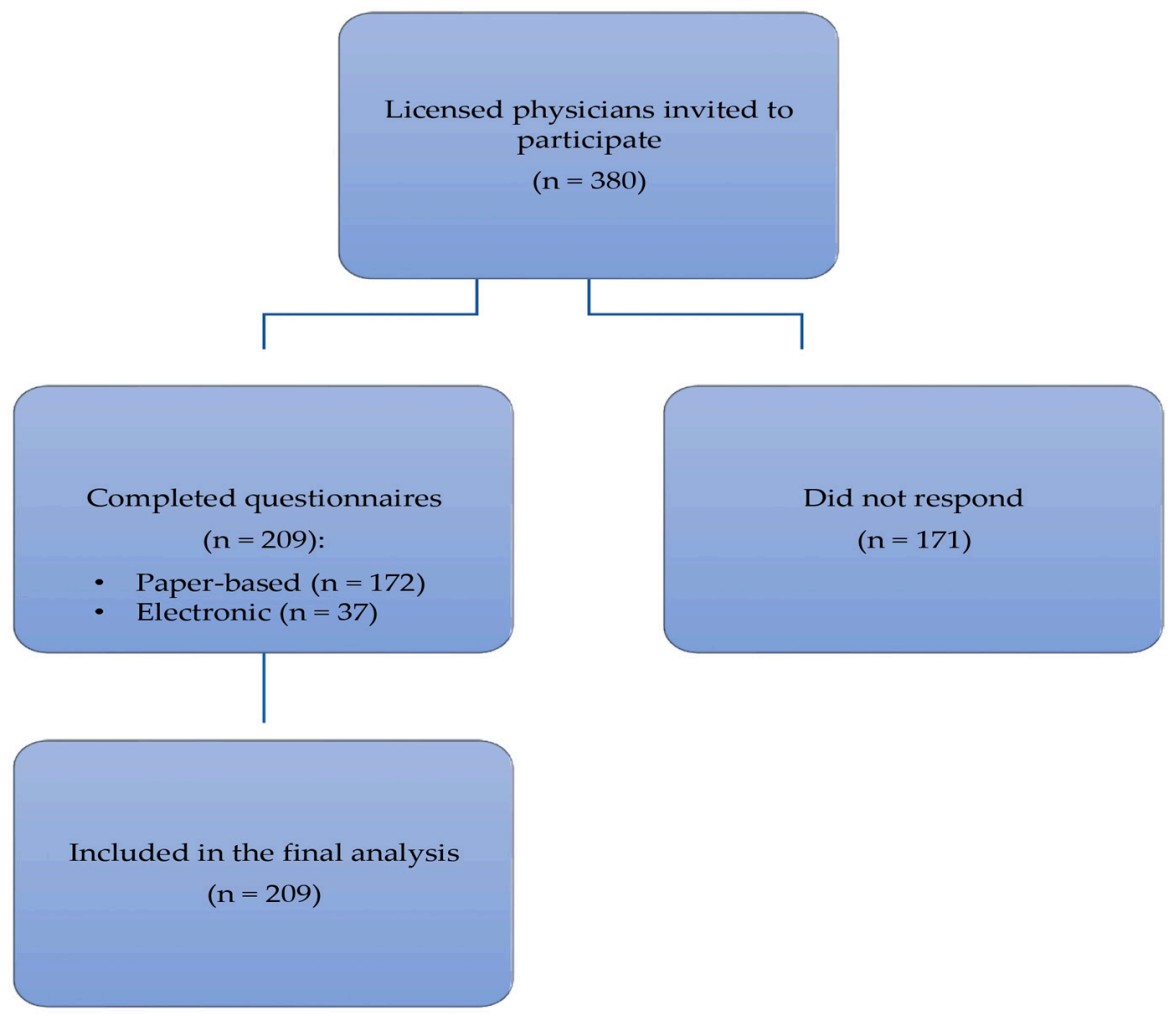
Flow diagram of participant recruitment and inclusion.

A total of 380 physicians were directly invited to participate. The questionnaire was completed by 209 physicians, yielding a response rate of 55.0%. Of the completed questionnaires, 172 were paper-based and 37 were completed electronically. The participant recruitment and inclusion process is presented in Figure 1. Participants were predominantly male (84.2%, *n* = 176), and more than half were younger than 40 years of age (52.6%, *n* = 110). Nearly half of the physicians were GPs (47.8%, *n* = 100). Most physicians were employed in the private sector (92.8%, *n* = 194), and approximately one-third (29.7%, *n* = 62) had more than 20 years of professional experience. The demographic characteristics of the participants are presented in Table 2.

Cronbach’s alpha coefficients for the attitude (13 items; α = 0.910, 95% CI: 0.891–0.927), view (5 items; α = 0.891, 95% CI: 0.866–0.913), and perception (10 items; α = 0.841, 95% CI: 0.807–0.872) scales were calculated, demonstrating excellent internal consistency for the attitude scale and good internal consistency for the view and perception scales. Detailed item-level internal consistency statistics, including corrected item–total correlations and Cronbach’s alpha if an item was deleted, are provided in Supplementary Tables S1–S3.

### 3.2. Extent of Collaboration and Communication with CPs

**Figure 2:**
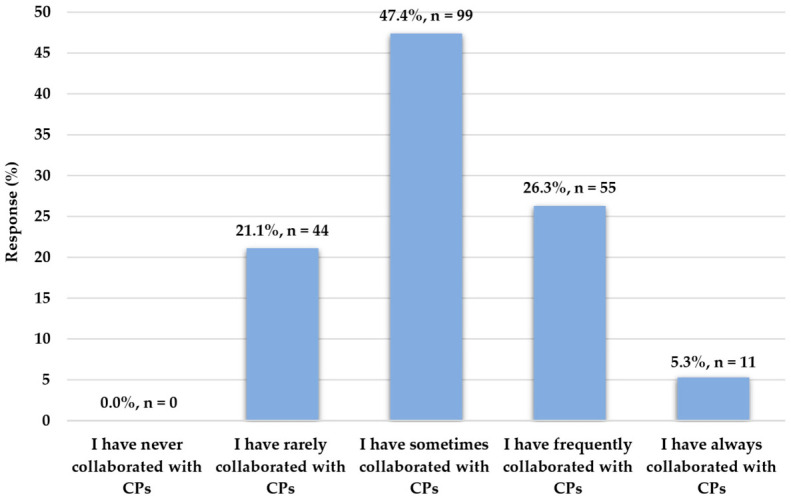
Frequency of collaboration between physicians and community pharmacists (CPs).

All physicians reported previous collaboration with CPs (Figure 2). Almost half of the participants indicated that collaboration with CPs occurred sometimes (47.4%, *n* = 99), whereas 26.3% (*n* = 55) reported frequent collaboration. In contrast, 21.1% (*n* = 44) of participants reported rarely collaborating with CPs. A minority reported always collaborating with CPs (5.3%, *n* = 11).

**Figure 3:**
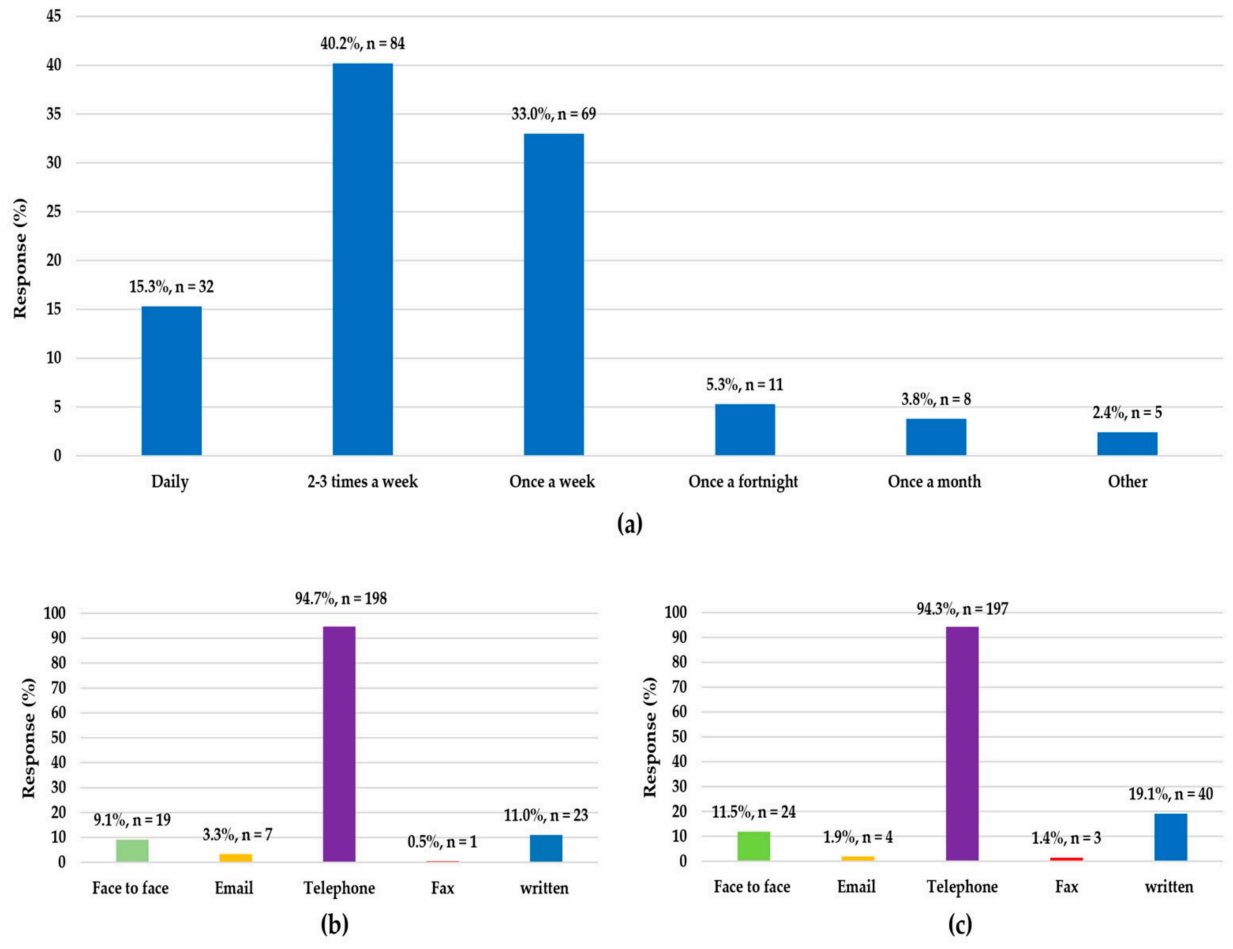
(**a**) Frequency of communication between physicians and community pharmacists (CPs). (**b**) The most common method(s) of communication between physicians and CPs. (**c**) The most preferred method(s) of communication between physicians and CPs. Participants could select more than one communication method in (**b**,**c**); therefore, percentages do not sum to 100%.

The most commonly reported frequencies of interaction with CPs were two to three times per week (40.2%, *n* = 84) and once per week (33.0%, *n* = 69) (Figure 3a). Additionally, the majority of physicians (94.7%, *n* = 198) reported that telephone was the most common and preferred method of communication between physicians and CPs (Figure 3b,c).

Physicians most often communicated with CPs to obtain advice on selecting the most appropriate medication for the patient (28.2%, *n* = 59), followed by improving patient adherence (27.8%, *n* = 58) and issues related to patients’ health insurance (26.8%, *n* = 56) (Table 3). In contrast, from the participants’ perspective, the most common reason for CPs to communicate with physicians was to verify prescribed medications and doses (85.6%, *n* = 179) (Table 4).

**Figure 4:**
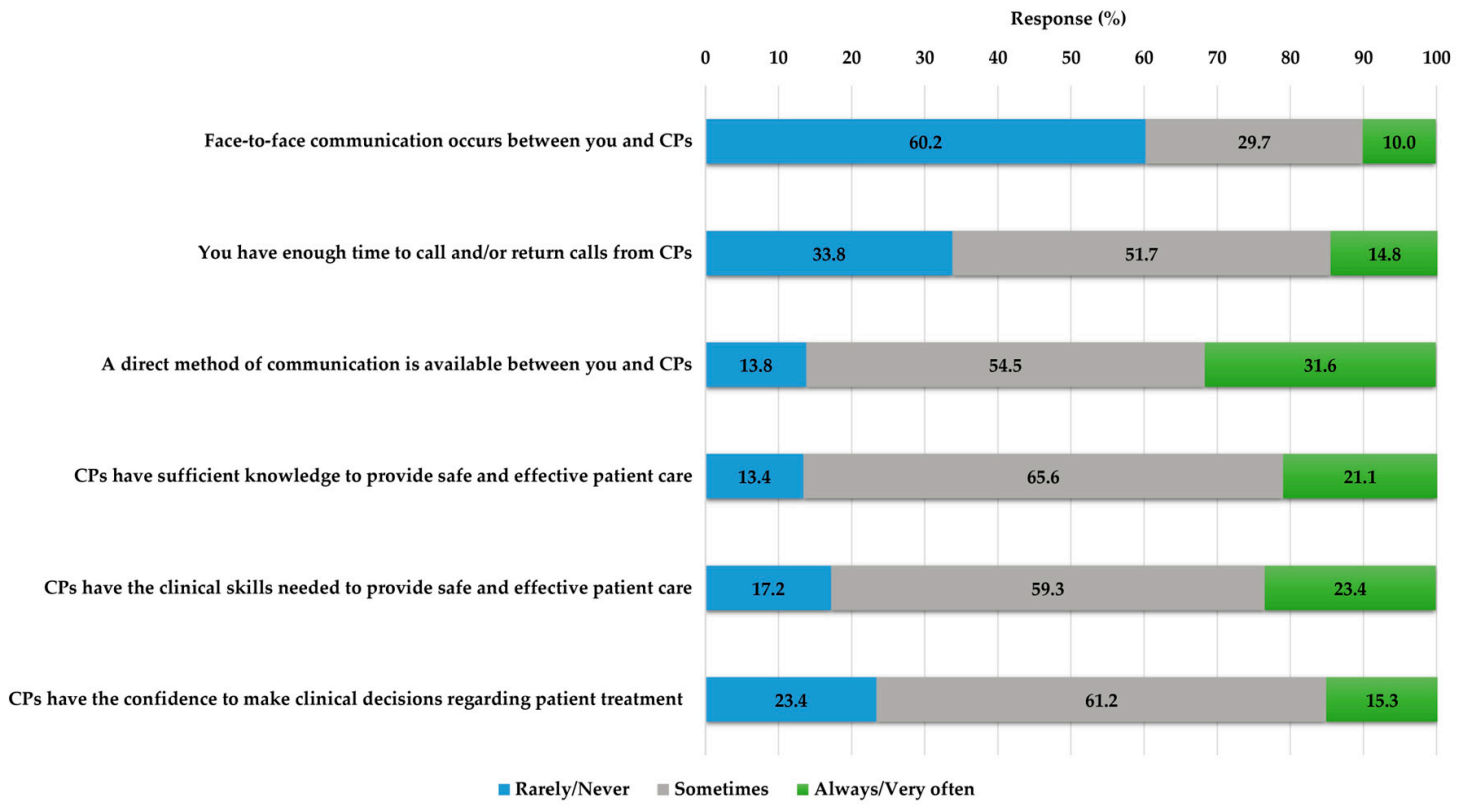
Frequency of issues encountered by the physicians when dealing with community pharmacists (CPs).

The frequency of issues encountered by physicians when dealing with CPs was also explored (Figure 4). More than half of the physicians indicated that face-to-face communication with CPs rarely or never occurred (60.2%, *n* = 126). Additionally, 51.7% of physicians (*n* = 108) reported only sometimes having sufficient time to contact CPs or return their calls. Furthermore, physicians perceived that CPs only sometimes had adequate clinical skills (59.3%, *n* = 124), confidence (61.2%, *n* = 128), or knowledge (65.6%, *n* = 137) to provide safe and effective patient care.

### 3.3. Physicians’ Attitudes Towards Collaboration with CPs

Most physicians expressed positive attitudes towards collaboration with CPs, with agreement rates exceeding 74.0% for seven of the 13 attitude statements (Table 5). The highest levels of agreement were observed for open communication (88.0%, *n* = 184), mutual respect (87.6%, *n* = 183), and shared goals in patient care (81.3%, *n* = 170). However, neutral attitudes were reported regarding trust in CPs’ decisions (42.1%, *n* = 88), therapeutic expertise (46.9%, *n* = 98), and their ability to meet professional expectations (33.0%, *n* = 69).

### 3.4. Physicians’ Views on the Impact of Collaboration on Patient Care

Despite varying levels of current collaboration, physicians believed that greater collaboration could positively influence patient outcomes and healthcare system efficiency (Table 6). Most respondents agreed or strongly agreed that collaboration between physicians and CPs could positively impact patient outcomes (98.1%, *n* = 205), reduce medication errors (97.1%, *n* = 203), improve continuity of patient care (89.0%, *n* = 186), and help alleviate pressure on the healthcare system (95.7%, *n* = 200). In addition, many physicians believed that collaboration with CPs could support physicians’ knowledge and confidence regarding medications (78.5%, *n* = 164).

### 3.5. Physicians’ Perceptions of the Professional Roles of CPs

Physicians generally expressed positive perceptions towards the professional roles of CPs. The highest level of agreement was reported for helping improve patient adherence (91.9%, *n* = 192), followed by dispensing prescriptions (89.0%, *n* = 186) and counselling patients about their prescribed medications (85.6%, *n* = 179) (Table 7).

**Figure 5:**
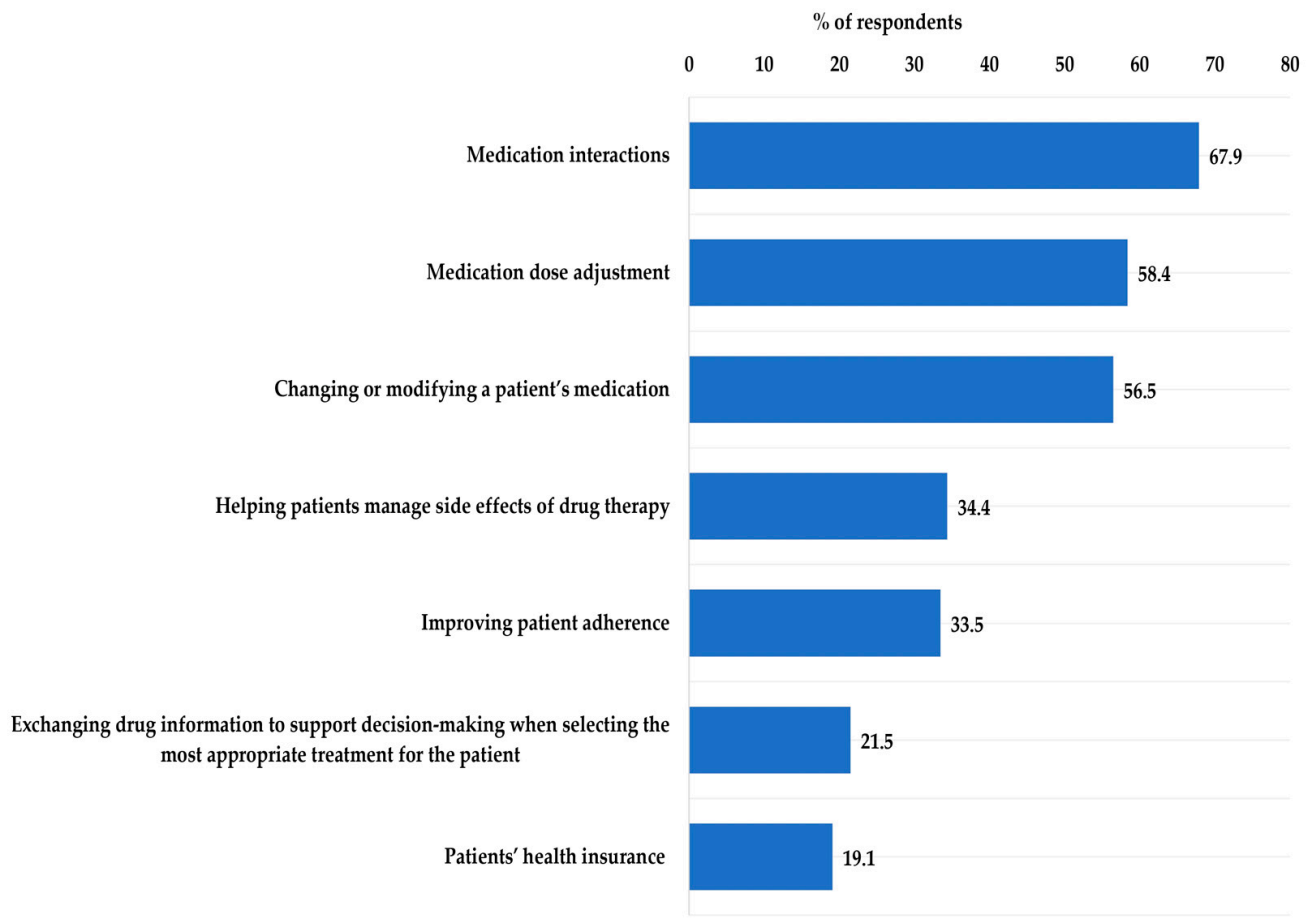
Physicians’ perceptions of areas for further collaboration between physicians and community pharmacists (CPs).

Physicians were asked to identify potential areas for future collaboration with CPs, as presented in Figure 5. Medication-related issues emerged as the main areas for potential future collaboration with CPs. The most frequently reported area was medication interactions (67.9%, *n* = 142).

### 3.6. Free-Text Comments

The comments provided by 63 physicians at the end of the questionnaire highlighted several suggestions for improving collaboration with CPs. The main suggestions included enhancing communication through regular meetings and conferences, conducting joint training sessions, and increasing awareness of the professional roles of both physicians and CPs:

“Through continuous meetings, relationships and communication between them can be improved” (PHY82).

“Pharmacists and physicians must communicate clearly and regularly to ensure that patients receive the best possible care” (PHY164).

“Increase awareness about the roles of each of them [physicians and CPs]” (PHY194).

### 3.7. Associations Between Physicians’ Demographic Characteristics and Attitude, View, and Perception Scores Towards Collaboration with CPs

Median attitude, view, and perception scores were generally high across participant subgroups, demonstrating overall positive attitudes, views, and perceptions towards collaboration with CPs (Table 8). No statistically significant differences in attitude, view, or perception scores were found in relation to gender (attitude: *p* = 0.357, view: *p* = 0.568, perception: *p* = 0.216), age (attitude: *p* = 0.569, view: *p* = 0.621, perception: *p* = 0.238), professional experience (attitude: *p* = 0.080, view: *p* = 0.669, perception: *p* = 0.432), or employment sector (attitude: *p* = 0.545, view: *p* = 0.699, perception: *p* = 0.323). However, significant differences were observed according to physicians’ job description for both attitude scores (*p* = 0.035, ε^2^ = 0.027) and perception scores (*p* = 0.025, ε^2^ = 0.031), with both analyses indicating small effect sizes. Post hoc pairwise comparisons with adjustment for multiple comparisons did not identify statistically significant differences between the individual job description categories for attitude scores. For perception scores, GPs had significantly higher perception scores than specialists (adjusted *p* = 0.014), whereas no other pairwise comparisons were statistically significant. No significant differences in view scores were identified based on job description (*p* = 0.719).

## 4. Discussion

This study investigated physicians’ experiences and attitudes towards collaboration with CPs in Jordan, as well as their views on the impact of such collaboration on patient care and their perceptions of CPs’ professional roles. Most participating physicians reported some degree of collaboration with CPs, although its frequency varied among participants. Notably, no participating physician reported never collaborating with a CP. This finding may partly reflect the definition of collaboration provided in the questionnaire, which encompassed cooperative working in patient care, together with the routine medication-related interactions between physicians and CPs, such as prescription clarification, medication-related enquiries, and patient-related consultations, which were commonly reported in the present study. These findings suggest that physicians and CPs interact and communicate regularly as part of routine clinical practice in Jordan. Although communication is an important component of collaborative care, it alone does not necessarily represent formal interprofessional collaboration, which typically involves shared decision-making and coordinated patient management. Therefore, the findings of the present study indicate opportunities to strengthen collaborative practice in Jordan, while also suggesting that formal collaborative practice models involving shared decision-making and coordinated patient management are not yet well established in primary care settings. Similar findings have been reported in a recent systematic review, which found that physician–pharmacist collaboration was usually informal and mainly reliant on individual professional relationships and communication practices rather than structured collaborative frameworks [18]. In contrast, more formal collaborative practice models have been implemented in several countries. In the United States, collaborative practice agreements represent a formal mechanism for physician–pharmacist collaboration, and pharmacist engagement under these agreements has been associated with improved clinical and financial outcomes and high provider satisfaction [32]. Similarly, a longitudinal study conducted in Spain found that implementing comprehensive medication review services was associated with increased collaboration between CPs and physicians over a 12-month period [33], while pharmacist-led primary care clinics in Canada support collaborative medication management within multidisciplinary healthcare teams [34].

Participating physicians identified the telephone as the most common and preferred method of communication with CPs, reflecting its convenience and practicality in routine clinical settings. In contrast, face-to-face communication appeared to be less common, which is relevant to the Jordanian healthcare system, where physicians and CPs usually work in separate practice settings [21]. The communication patterns observed in this study may indicate a need for more structured opportunities for interprofessional communication in Jordan. Similar communication barriers have been reported internationally, where the lack of integrated communication systems has limited effective physician–pharmacist collaboration [19,35]. Improving communication between the two professions may enhance medication-related decision-making, strengthen professional relationships, reduce fragmentation in medication management services, and facilitate continuity of care [2,15,18,36].

In the present study, physicians indicated the frequency of issues they encountered when dealing with CPs. Time constraints emerged as one of the main barriers affecting communication between physicians and CPs. These findings may reflect the heavy workload and time pressures experienced by physicians in Jordanian healthcare settings. A cross-sectional study found that work–life conflict and workload-related stress are highly prevalent among Jordanian physicians [37] and may negatively affect communication and collaboration with other HCPs. The identified time and communication barriers align with findings from previous studies conducted internationally and in other Arab countries, including Kuwait, the United Arab Emirates, and Iraq, and highlight the need for unified communication platforms and formalised collaboration pathways to facilitate routine physician–pharmacist communication and support collaborative medication management [2,15,25,35].

Some uncertainty regarding the clinical role of CPs was also observed among participating physicians, which may indicate a lack of role clarity between physicians and CPs. Physicians indicated that CPs only sometimes possessed sufficient clinical knowledge, clinical skills, and confidence to make clinical decisions regarding patient treatment. This may also reflect the current scope of community pharmacy practice in Jordan, where pharmacists’ roles have traditionally focused on medication dispensing and counselling, while the integration of broader pharmaceutical-care services into primary healthcare remains under development [38]. CPs in Jordan do not have prescribing authority [21], lack routine access to patients’ medical records [39], and structured medication review services are not routinely implemented in community pharmacies [40]. These characteristics may provide context for the uncertainty expressed by participating physicians regarding pharmacists’ therapeutic decision-making and clinical expertise. In the free-text comments, participating physicians emphasised the need for regular meetings, joint training sessions, and improved communication between physicians and CPs. Such initiatives may enhance understanding of the roles and competencies of CPs, improve physicians’ confidence in pharmacists’ clinical roles [18,41], and promote greater role clarity between the two professions. Those recommendations have been reported in Jordan and other Arab countries, including Kuwait, Qatar, and the United Arab Emirates, as well as internationally as important facilitators of sustainable interprofessional collaboration [2,15,26,33,34,39,41,42,43,44].

Positive attitudes towards communication, mutual respect, and shared goals were identified in this study, which may highlight physicians’ readiness for collaborative practice with CPs. However, the neutral attitudes towards CPs’ clinical expertise and decision-making abilities indicate ongoing uncertainty about CPs’ professional roles and competencies. Similar findings have been reported internationally, where physicians acknowledged the value of pharmacists in patient care while showing uncertainties about their clinical responsibilities, therapeutic expertise, and participation in decision-making processes [45,46]. Previous literature from Arab countries and other international settings has shown that unclear professional roles and limited interprofessional interaction may negatively affect trust and collaborative relationships between HCPs and remain important barriers to effective interprofessional collaboration in both high-income and middle-income healthcare settings [2,15,18,29,36].

Physicians expressed overwhelmingly positive views regarding the potential benefits of collaboration with CPs, particularly in improving patient outcomes, medication safety, and healthcare delivery. Together with the reported collaborative experiences, these findings indicate that physicians perceive greater collaboration with CPs as having the potential to improve patient care and promote safer medication use. This observation is important because medication errors are one of the most common and preventable sources of patient harm [47]. In addition, most participants agreed that collaboration between physicians and CPs could help reduce pressure on the healthcare system. These physicians’ perceptions are consistent with previous studies reporting that pharmacist involvement in collaborative care has been associated with reduced physician workload and enhanced delivery of healthcare services [48]. Many physicians also believed that collaboration with CPs could support physicians’ knowledge and confidence regarding medications. CPs are medication experts and may provide physicians with valuable medication-related information, including advice regarding medication availability, medication interactions, and appropriate medication use [41].

Regarding physicians’ perceptions of CPs’ professional roles, the role of CPs in improving patient adherence was the most strongly recognised. Medication adherence remains a major challenge across healthcare systems worldwide, particularly among patients with chronic diseases [49,50]. CPs are among the most accessible HCPs, providing opportunities to support medication management. Previous studies have shown that pharmacist-led interventions may improve medication adherence through patient counselling, medication education, follow-up, and identification of medication-related problems [51]. Physicians also identified medication-related issues as important areas for potential future collaboration with CPs. The most commonly reported area was medication interactions. Expanding collaborative practice between physicians and CPs in these medication-related areas may support better patient outcomes and safer medication use within the Jordanian healthcare system.

Significant overall differences in attitude and perception scores were identified according to physicians’ job description. However, post hoc pairwise comparisons did not identify statistically significant differences between the individual job description categories for attitude scores. For perception scores, GPs demonstrated significantly higher perception scores than specialists. Although statistically significant overall differences were observed across the job description categories, the effect sizes were small, and these subgroup findings should be interpreted with caution. A possible explanation for this finding is that GPs may be more likely to encounter patients with chronic diseases and polypharmacy, potentially providing more opportunities for communication and collaboration with CPs [33,52]. Future studies are needed to investigate this possibility.

The findings of this study have several implications for healthcare policy and practice in Jordan. Developing collaborative practice frameworks, clearly defining the professional roles and responsibilities of physicians and CPs, strengthening interprofessional education and training, establishing formal communication pathways, and expanding pharmacists’ participation in medication management services may help strengthen physician–pharmacist collaboration. Such initiatives may facilitate greater integration of CPs into primary healthcare, promote collaborative patient care, and ultimately improve patient outcomes.

### Strengths and Limitations

This is the first study, to our knowledge, to examine physicians’ perspectives on collaboration with CPs in Jordan. The study included physicians with various professional backgrounds and levels of experience. Compared with national physician workforce data, the gender distribution of the study sample was broadly consistent with national estimates. Male physicians represented 84.2% of the present sample, compared with 83.1% among physicians employed by the Jordanian Ministry of Health [53]. Furthermore, physicians are concentrated in Amman, which has the highest physician density among Jordanian governorates [53]. However, the proportion of physicians employed in the private sector was higher in the present sample than that reported nationally (92.8% versus approximately 64%) [22].

Despite these strengths, this study has some limitations. The study findings may not be generalisable to all physicians practising in Jordan because the study was conducted only among physicians practising in Amman, the achieved sample size was lower than the calculated target sample size, and convenience sampling may have introduced selection bias. Nevertheless, several findings were in agreement with those reported in previous international studies on this topic [2,4,16,17,18,19]. Formal psychometric validation of the newly developed questionnaire sections (Supplementary Sections D and E) was not undertaken. However, these sections were developed based on an extensive literature review and pilot tested to assess their face validity and clarity prior to data collection. Furthermore, a validated scale (ATCI-GP) was used to measure attitudes towards collaboration. The analyses were exploratory and did not adjust for potential confounding variables; therefore, the observed associations should be interpreted with caution. Future studies employing multivariable modelling are warranted to identify factors independently associated with physicians’ attitudes, views, and perceptions towards collaboration with CPs. As the data were self-reported, the findings may be subject to recall bias and social desirability bias. However, the use of an anonymous questionnaire may have reduced social desirability bias.

## 5. Conclusions

The physicians participating in this study reported previous experiences of collaboration with CPs, although regular collaboration was not consistently reported among all participants. Overall, they demonstrated positive attitudes towards collaboration with CPs. However, the uncertainty regarding CPs’ clinical roles and decision-making abilities emphasises the need for clearer role definition, improved interprofessional communication, and greater awareness of CPs’ clinical competencies. As this study assessed physicians’ self-reported perceptions, it did not evaluate the impact of physician–CP collaboration on clinical or healthcare outcomes. These findings may inform policymakers, healthcare institutions, and professional associations in Jordan in developing strategies to strengthen collaboration between physicians and CPs. However, the findings should be interpreted in the context of a convenience sample of physicians practising in Amman, predominantly in the private sector, and should not be generalised to all physicians in Jordan. Future research should explore CPs’ perspectives and prospectively assess the impact of structured collaborative practice models on patient care and healthcare delivery.

## Figures and Tables

**Table 1:** Questionnaire sections.

Section	Description
Supplementary Section A	Demographic characteristics of physicians and details of their working environment.
Supplementary Section B	Physicians’ experiences of collaboration with CPs, including communication frequency, reasons for contact, and barriers to collaboration.
Supplementary Section C	Physicians’ attitudes towards collaboration with CPs measured using the ATCI-GP.
Supplementary Section D	Physicians’ views on collaboration with CPs and its impact on patient care.
Supplementary Section E	Physicians’ perceptions of the professional roles of CPs. The section ended with an open-ended question inviting additional comments on interprofessional collaboration.

**Table 2:** Demographic characteristics of participants (*N* = 209).

Characteristics	*N* (%) or Median (IQR)
Gender	
Female	33 (15.8)
Male	176 (84.2)
Age (years)	
24–30	55 (26.3)
31–39	55 (26.3)
40–49	39 (18.7)
50–59	38 (18.2)
≥60	22 (10.5)
Job description	
General practitioner	100 (47.8)
Resident	21 (10.0)
Specialist	40 (19.1)
Consultant	48 (23.0)
Years of professional experience	
≤5	56 (26.8)
6–10	45 (21.5)
11–20	46 (22.0)
>20	62 (29.7)
Employment Sector	
Private sector	194 (92.8)
Public sector	15 (7.2)

**Table 3:** Reasons for physicians to communicate with community pharmacists (CPs).

Reasons for Physicians to Communicate with CPs	*N* (%)
Choosing the most appropriate medication for the patient	59 (28.2)
Improving patient adherence	58 (27.8)
Patients’ health insurance issues	56 (26.8)
Modifying a patient’s medication regimen	52 (24.9)
Medication dose adjustments	42 (20.1)
Managing side effects of medication	29 (13.9)
Medication interactions	18 (8.6)

Note. Participants could select more than one option; therefore, percentages do not sum to 100%.

**Table 4:** Reasons for community pharmacists (CPs) to communicate with physicians.

Reasons for CPs to Communicate with Physicians	*N* (%)
Clarification of prescribed medications and doses	179 (85.6)
Answering patient-related inquiries	107 (51.2)
Recommendations to change a patient’s medication	105 (50.2)
Patients’ health insurance issues	65 (31.1)
Medication dose adjustments	51 (24.4)
Advice regarding medication side effects	32 (15.3)
Medication interactions	12 (5.7)
Improving patient adherence	12 (5.7)

Note. Participants could select more than one option; therefore, percentages do not sum to 100%.

**Table 5:** Physicians’ attitudes towards collaboration with community pharmacists (CPs).

Statement	Strongly Disagree/Disagree *N* (%)	Neither Agree nor Disagree *N* (%)	Agree/Strongly Agree *N* (%)
1. The professional communication between myself and the pharmacist is open and honest.	14 (6.7)	11 (5.3)	184 (88.0)
2. The pharmacist is open to working together with me on patients’ medication management.	9 (4.3)	44 (21.1)	156 (74.6)
3. The pharmacist delivers high-quality healthcare to patients.	21 (10.0)	75 (35.9)	113 (54.1)
4. The pharmacist has time to discuss with me matters relating to patients’ medication regimens.	12 (5.7)	75 (35.9)	122 (58.4)
5. The pharmacist meets the professional expectations I have of him/her.	41 (19.6)	69 (33.0)	99 (47.4)
6. I can trust the pharmacist’s professional decisions.	23 (11.0)	88 (42.1)	98 (46.9)
7. The pharmacist actively addresses patients’ medical concerns.	27 (12.9)	74 (35.4)	108 (51.7)
8. The pharmacist and I have mutual respect for one another on a professional level.	5 (2.4)	21 (10.0)	183 (87.6)
9. The pharmacist and I share common goals and objectives when caring for the patient.	4 (1.9)	35 (16.7)	170 (81.3)
10. My role and the pharmacist’s role in patient care are clear.	13 (6.2)	30 (14.4)	166 (79.4)
11. I have confidence in the pharmacist’s expertise in medicines and therapeutics.	15 (7.2)	98 (46.9)	96 (45.9)
12. The pharmacist has a role in assuring medication safety (for example, to identify drug interactions, adverse reactions, contraindications, etc.).	14 (6.7)	35 (16.7)	160 (76.6)
13. The pharmacist has a role in assuring medication effectiveness (for example, to ensure the patient receives the optimal drug at the optimal dose, etc.).	9 (4.3)	33 (15.8)	167 (79.9)

**Table 6:** Physicians’ views on the impact of collaboration with community pharmacists (CPs).

Statement	Strongly Disagree/Disagree *N* (%)	Neither Agree nor Disagree *N* (%)	Agree/Strongly Agree *N* (%)
1. Collaboration between physicians and community pharmacists could positively impact on patient outcomes.	2 (1.0)	2 (1.0)	205 (98.1)
2. Collaboration between physicians and community pharmacists could help reduce medication errors.	2 (1.0)	4 (1.9)	203 (97.1)
3. Collaboration between physicians and community pharmacists could improve continuity of patient care.	5 (2.4)	18 (8.6)	186 (89.0)
4. Collaboration between physicians and community pharmacists could help reduce pressure on the healthcare system.	2 (1.0)	7 (3.3)	200 (95.7)
5. Collaboration between physicians and community pharmacists could support physicians’ knowledge and confidence regarding medications.	18 (8.6)	27 (12.9)	164 (78.5)

**Table 7:** Physicians’ perceptions of the professional roles of community pharmacists (CPs).

Professional Role of CPs	Strongly Disagree/Disagree *N* (%)	Neither Agree nor Disagree *N* (%)	Agree/Strongly Agree *N* (%)
Assisting patients in selecting over-the-counter medications	32 (15.3)	14 (6.7)	163 (78.0)
Counselling patients about their prescribed medications	15 (7.2)	15 (7.2)	179 (85.6)
Helping patients manage medication side effects	6 (2.9)	34 (16.3)	169 (80.9)
Providing advice regarding medication interactions	4 (1.9)	35 (16.7)	170 (81.3)
Helping improve patient adherence	7 (3.3)	10 (4.8)	192 (91.9)
Providing medication information to physicians to support medication selection	28 (13.4)	43 (20.6)	138 (66.0)
Providing advice on managing medication-related problems	27 (12.9)	36 (17.2)	146 (69.9)
Dispensing prescriptions	13 (6.2)	10 (4.8)	186 (89.0)
Assisting with medication dose adjustments	10 (4.6)	43 (20.6)	156 (74.6)
Assisting with issues related to patients’ health insurance, including obtaining insurance company approvals	4 (1.9)	34 (16.3)	171 (81.8)

**Table 8:** Associations between physicians’ demographic characteristics and attitude, view, and perception scores towards collaboration with community pharmacists (CPs).

Variable	Median Attitude Score (IQR)	*p*-Value	Median View Score (IQR)	*p*-Value	Median Perception Score (IQR)	*p*-Value
Gender						
Male	3.69 (0.69)	0.357	4.00 (0.50)	0.568	3.90 (0.40)	0.216
Female	3.69 (0.54)		4.00 (0.88)		4.00 (0.45)	
Age						
<40	3.73 (0.63)	0.569	4.00 (1.00)	0.621	4.00 (0.40)	0.238
≥40	3.62 (0.69)		4.00 (0.50)		3.90 (0.50)	
Job description						
General practitioner	3.85 (0.54)	**0.035**	4.00 (0.50)	0.719	4.00 (0.30)	**0.025**
Resident	3.69 (0.92)		4.00 (0.25)		4.00 (0.40)	
Specialist	3.42 (0.77)		4.00 (1.25)		3.70 (0.68)	
Consultant	3.54 (0.52)		4.00 (0.50)		3.95 (0.50)	
Professional experience in years						
≤5	3.69 (0.83)	0.080	4.00 (1.00)	0.669	4.00 (0.40)	0.432
6–10	4.00 (0.46)		4.00 (0.50)		3.90 (0.35)	
11–20	3.58 (0.77)		4.00 (0.63)		3.90 (0.60)	
>20	3.58 (0.69)		4.00 (0.50)		4.00 (0.50)	
Employment sector						
Private sector	3.69 (0.69)	0.545	4.00 (0.50)	0.699	4.00 (0.40)	0.323
Public sector	3.53 (0.77)		4.00 (0.50)		3.60 (0.70)	

Note. Statistically significant *p*-values are shown in bold. Mann–Whitney U tests were used for comparisons between two groups (gender, age, and employment sector), whereas Kruskal–Wallis tests were used for comparisons involving more than two groups (job description and professional experience).

## Data Availability

The data presented in this study are available from the corresponding author upon reasonable request. The data are not publicly available due to ethical and privacy considerations.

## Supplementary Materials

The following supporting information can be downloaded at: https://www.mdpi.com/article/10.3390/healthcare14152335/s1, File S1: Physician Questionnaire; Table S1: Item-level internal consistency of the attitudes scale; Table S2: Item-level internal consistency of the views scale; Table S3: Item-level internal consistency of the perceptions scale.

## Abbreviations

The following abbreviations are used in this manuscript:

ATCI-GP	Attitudes Towards Collaboration Instrument for GPs
CP	Community pharmacist
GP	General practitioner
HCP	Healthcare professional
IQR	Interquartile range
