# Supplementary material for: Physicians’ Perspectives on Collaboration with Community Pharmacists: Implications for Collaborative Care in Jordan
Source: Healthcare (Basel). 2026 Aug 1;14(15):2335. doi: 10.3390/healthcare14152335 (PMC13465426; doi:10.3390/healthcare14152335)
Supplement: Supplementary file 1 [file healthcare-14-02335-s001.zip › healthcare-4387653-supplementary.pdf]

Supplementary Material

File S1. Physician Questionnaire

Physicians' perceptions toward collaboration with community pharmacists in Jordan: a cross-sectional survey

Interprofessional collaboration has been described as "cooperatively working together, sharing responsibilities for solving problems and making decisions to formulate and carry out plans for patient care". The purpose of this study is to explore physicians' experiences of collaboration with community pharmacists in Jordan, as well as their attitudes, views, and perceptions regarding such collaboration.

HOW TO COMPLETE THIS QUESTIONNAIRE:

This questionnaire will take approximately 10 minutes to complete.
Participation is voluntary, and all responses will be anonymous and treated confidentially.
No identifying information will be collected, and the information provided will be used solely for research purposes. There are no right or wrong answers; we are interested in your views and experiences regarding collaboration with community pharmacists.

Section A	Demographic information
Section B	Collaboration and communication with community pharmacists
Section C	Attitudes towards collaboration with community pharmacists
Section D	Views on collaboration with community pharmacists and its impact on patient care.
Section E	Perceptions of the professional roles of community pharmacists

SECTION A: DEMOGRAPHIC INFORMATION

1. **Gender**

Female ☐ Male ☐

2. **Age (years)**

24–30 ☐ 31–39 ☐ 40–49 ☐ 50–59 ☐ ≥60 ☐

3. **Job Description**

General Practitioner ☐ Resident ☐ Specialist ☐ Consultant ☐

4. **Years of Professional Experience**

≤5 ☐ 6-10 ☐ 11-20 ☐ >20 ☐

5. **Number of hours working per week: ____________________________**

6. **Employment Sector**

Private Sector ☐ Public Sector ☐

SECTION B: COLLABORATION AND COMMUNICATION WITH COMMUNITY PHARMACISTS

7. How often have you collaborated with community pharmacists?

- I have **never** collaborated with community pharmacists (Continue to Q8; skip Section C) ☐
- I have **rarely** collaborated with community pharmacists ☐
- I have **sometimes** collaborated with community pharmacists ☐
- I have **frequently** collaborated with community pharmacists ☐
- I have **always** collaborated with community pharmacists ☐

8. On average, how often would you be in contact with community pharmacists?

Never (Go to Q14; skip Section C)	Daily	2-3 times/week	Once/week	Once/2 weeks	Once/month	Other:
☐	☐	☐	☐	☐	☐	__________

9. What is/are the most common method(s) of communication between you and community pharmacists? Please tick all that apply:

Face-to-face	Telephone	Email	Written	Fax	Other:
☐	☐	☐	☐	☐	__________

10. What is/are the preferred method(s) of communication between you and the community pharmacists? Please tick all that apply:

Face-to-face	Telephone	Email	Written	Fax	Other:
☐	☐	☐	☐	☐	__________

11. **What are the most <u>common reasons</u> for the <u>community pharmacists</u> to communicate with you? Please tick all that apply:**

- Recommendations to change a patient's medication ☐
- Medication dose adjustments ☐
- Answering patient-related inquiries ☐
- Medication interactions ☐
- Improving patient adherence ☐
- Clarification of prescribed medications and doses ☐
- Advice regarding medication side effects ☐
- Patients' health insurance issues ☐
- Other: ______________________________ ☐

12. **What are the most <u>common reasons</u> for <u>you</u> to communicate with the community pharmacists? Please tick all that apply:**

- Modifying a patient's medication regimen ☐
- Medication dose adjustments ☐
- Managing side effects of medication ☐
- Choosing the most appropriate medication for the patient ☐
- Improving patient adherence ☐
- Medication interactions ☐
- Patients' health insurance issues ☐
- Other: ______________________________ ☐

13. Using the scale provided, please rate how often you encounter the following when dealing with community pharmacists, by placing a tick (✓) in the appropriate box:

	Always	Very often	Sometimes	Rarely	Never
• Face-to-face communication occurs between you and community pharmacists	☐	☐	☐	☐	☐
• You have enough time to call and/or return calls from community pharmacists	☐	☐	☐	☐	☐
• A direct method of communication is available between you and community pharmacists	☐	☐	☐	☐	☐
• Community pharmacists have sufficient knowledge to provide safe and effective patient care	☐	☐	☐	☐	☐
• Community pharmacists have the clinical skills needed to provide safe and effective patient care	☐	☐	☐	☐	☐
• Community pharmacists have the confidence to make clinical decisions regarding patient treatment	☐	☐	☐	☐	☐

14. In your opinion, what are the <u>potential areas</u> for <u>further collaboration</u> between physicians and community pharmacists in Jordan? Please tick all that apply:

- Improving patient adherence ☐
- Helping patients manage side effects of drug therapy ☐
- Medication interactions ☐
- Exchanging drug information to support decision-making when selecting the most appropriate treatment for the patient ☐
- Medication dose adjustment ☐
- Changing or modifying a patient’s medication ☐
- Patients’ health insurance ☐
- Other: ______________________________ ☐

SECTION C: ATTITUDES TOWARDS COLLABORATION WITH COMMUNITY PHARMACISTS

Please indicate the extent to which you agree or disagree with the following statements by placing a tick (✓) in the appropriate box:

	Strongly disagree	Disagree	Neither agree nor disagree	Agree	Strongly agree
**15. The professional communication between myself and the pharmacist is open and honest**	☐	☐	☐	☐	☐
**16. The pharmacist is open to working together with me on patients' medication management**	☐	☐	☐	☐	☐
**17. The pharmacist delivers high quality healthcare to patients**	☐	☐	☐	☐	☐
**18. The pharmacist has time to discuss with me matters relating to patients' medication regimens.**	☐	☐	☐	☐	☐
**19. The pharmacist meets the professional expectations I have of him/her**	☐	☐	☐	☐	☐
**20. I can trust the pharmacist's professional decisions**	☐	☐	☐	☐	☐
**21. The pharmacist actively addresses patients' medical concerns**	☐	☐	☐	☐	☐
**22. The pharmacist and I have mutual respect on a professional level**	☐	☐	☐	☐	☐
**23. The pharmacist and I share common goals and objectives when caring for the patient**	☐	☐	☐	☐	☐
**24. My role and the pharmacist's role in patient care are clear**	☐	☐	☐	☐	☐

	Strongly disagree	Disagree	Neither agree nor disagree	Agree	Strongly agree
25. **I have confidence in the pharmacist's expertise in medicines and therapeutics**	☐	☐	☐	☐	☐
26. **The pharmacist has a role in assuring medication safety (for example, to identify drug interactions, adverse reactions, contraindications etc)**	☐	☐	☐	☐	☐
27. **The pharmacist has a role in assuring medication effectiveness (for example, to ensure the patient receives the optimal drug at the optimal dose etc)**	☐	☐	☐	☐	☐

SECTION D: VIEWS ON COLLABORATION WITH COMMUNITY PHARMACISTS AND ITS IMPACT ON PATIENT CARE

Please indicate the extent to which you agree or disagree with the following by placing a tick (✓) in the appropriate box:

	Strongly disagree	Disagree	Neither agree nor disagree	Agree	Strongly agree
**28. Collaboration between physicians and community pharmacists could positively impact on patient outcomes**	☐	☐	☐	☐	☐
**29. Collaboration between physicians and community pharmacists could help reduce medication errors**	☐	☐	☐	☐	☐
**30. Collaboration between physicians and community pharmacists could improve continuity of patient care**	☐	☐	☐	☐	☐
**31. Collaboration between physicians and community pharmacists could help reduce pressure on the healthcare system**	☐	☐	☐	☐	☐
**32. Collaboration between physicians and community pharmacists could support physicians' knowledge and confidence regarding medications**	☐	☐	☐	☐	☐

SECTION E: PERCEPTIONS OF THE PROFESSIONAL ROLES OF COMMUNITY PHARMACISTS

Please indicate the extent to which you agree or disagree with the following statements by placing a tick (✓) in the appropriate box:

	Strongly disagree	Disagree	Neither agree nor disagree	Agree	Strongly agree
**33. Assisting patients in selecting over-the-counter medications**	☐	☐	☐	☐	☐
**34. Counselling patients about their prescribed medications**	☐	☐	☐	☐	☐
**35. Helping patients manage medication side effects**	☐	☐	☐	☐	☐
**36. Providing advice regarding medication interactions**	☐	☐	☐	☐	☐
**37. Helping improve patient adherence**	☐	☐	☐	☐	☐
**38. Providing medication information to physicians to support medication selection**	☐	☐	☐	☐	☐
**39. Providing advice on managing medication-related problems**	☐	☐	☐	☐	☐
**40. Dispensing prescriptions**	☐	☐	☐	☐	☐
**41. Assisting with medication dose adjustments**	☐	☐	☐	☐	☐
**42. Assisting with issues related to patients' health insurance, including obtaining insurance company approvals**	☐	☐	☐	☐	☐

43. Do you have any additional comments about collaboration between physicians and community pharmacists in Jordan?

____

____

____

____

____

____

____

Thank you for taking the time to complete this questionnaire; your response is much appreciated.

Table S1. Item-level internal consistency of the attitudes scale

Item	Corrected item–total correlation	Cronbach's α if item deleted
1. The professional communication between myself and the pharmacist is open and honest	0.570	0.905
2. The pharmacist is open to working together with me on patients' medication management	0.577	0.905
3. The pharmacist delivers high quality healthcare to patients	0.700	0.900
4. The pharmacist has time to discuss with me matters relating to patients' medication regimens.	0.599	0.904
5. The pharmacist meets the professional expectations I have of him/her	0.714	0.899
6. I can trust the pharmacist's professional decisions	0.746	0.898
7. The pharmacist actively addresses patients' medical concerns	0.680	0.901
8. The pharmacist and I have mutual respect for one another on a professional level	0.561	0.906
9. The pharmacist and I share common goals and objectives when caring for the patient	0.642	0.903
10. My role and the pharmacist's role in patient care are clear	0.592	0.905
11. I have confidence in the pharmacist's expertise in medicines and therapeutics	0.750	0.898
12. The pharmacist has a role in assuring medication safety (for example, to identify drug interactions, adverse reactions, contraindications etc)	0.554	0.906
13. The pharmacist has a role in assuring medication effectiveness (for example, to ensure the patient receives the optimal drug at the optimal dose etc)	0.489	0.908

Table S2. Item-level internal consistency of the views scale

Item	Corrected item–total correlation	Cronbach's α if item deleted
1. Collaboration between physicians and community pharmacists could positively impact on patient outcomes	0.818	0.855
2. Collaboration between physicians and community pharmacists could help reduce medication errors	0.788	0.859
3. Collaboration between physicians and community pharmacists could improve continuity of patient care	0.802	0.851
4. Collaboration between physicians and community pharmacists could help reduce pressure on the healthcare system	0.828	0.851
5. Collaboration between physicians and community pharmacists could support physicians' knowledge and confidence regarding medications	0.585	0.924

Table S3. Item-level internal consistency of the perceptions scale

Item	Corrected item–total correlation	Cronbach's α if item deleted
Assisting patients in selecting over-the-counter medications	0.615	0.820
Counselling patients about their prescribed medications	0.506	0.830
Helping patients manage medication side effects	0.444	0.835
Providing advice regarding medication interactions	0.666	0.817
Helping improve patient adherence	0.514	0.830
Providing medication information to physicians to support medication selection	0.570	0.824
Providing advice on managing medication-related problems	0.639	0.816
Dispensing prescriptions	0.484	0.831
Assisting with medication dose adjustments	0.556	0.825
Assisting with issues related to patients' health insurance, including obtaining insurance company approvals	0.418	0.837
